# 2-[(2-Acet­oxy­benzo­yl)­oxy]benzoic acid

**DOI:** 10.1107/S1600536812026475

**Published:** 2012-06-16

**Authors:** Katarzyna A. Solanko, Andrew D. Bond

**Affiliations:** a University of Southern Denmark, Department of Physics, Chemistry and Pharmacy, Campusvej 55, 5230 Odense, Denmark

## Abstract

The title compound, C_16_H_12_O_6_, is a common impurity of *ortho*-acetyl­salicylic acid (aspirin). The benzene rings form a dihedral angle of 81.9 (1)° while the acetyl and carboxyl groups form dihedral angles of 74.0 (1) and 26.4 (2)°, respectively, with the benzene rings to which they are bound. In the crystal, mol­ecules are linked by pairs of O—H⋯O hydrogen bonds between the carboxyl groups, forming inversion dimers.

## Related literature

For background literature concerning the crystallization and crystal structure of aspirin, see: Bond *et al.* (2007 ▶, 2011 ▶). For a discussion of the pharmacological effects of acetyl­salicyl­salicylic acid, see: Bundgaard (1974 ▶). For related structures, see: Greener *et al.* (2000 ▶); Cox *et al.* (2000 ▶); Iqbal *et al.* (2007 ▶).
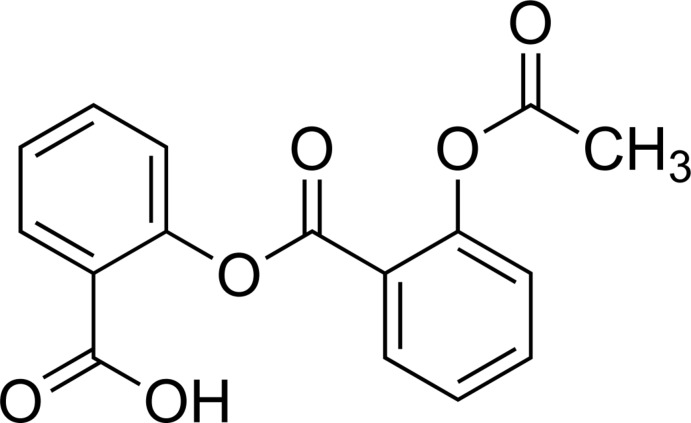


## Experimental

### 

#### Crystal data

C_16_H_12_O_6_
*M* *_r_* = 300.26
Monoclinic, 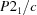

*a* = 9.6314 (5) Å
*b* = 7.7548 (3) Å
*c* = 18.0763 (8) Å
β = 95.572 (2)°
*V* = 1343.73 (11) Å^3^
*Z* = 4
Mo *K*α radiation
μ = 0.12 mm^−1^
*T* = 150 K
0.40 × 0.20 × 0.02 mm

#### Data collection

Bruker Nonius X8 APEXII CCD diffractometer
Absorption correction: multi-scan (*SADABS*; Sheldrick, 2003 ▶) *T*_min_ = 0.887, *T*_max_ = 0.998
15807 measured reflections
2367 independent reflections
1868 reflections with *I* > 2σ(*I*)
*R*_int_ = 0.031

#### Refinement

*R*[*F* ^2^ > 2σ(*F* ^2^)] = 0.033
*wR*(*F* ^2^) = 0.080
*S* = 1.04
2367 reflections
204 parameters
1 restraint
H atoms treated by a mixture of independent and constrained refinement
Δρ_max_ = 0.18 e Å^−3^
Δρ_min_ = −0.17 e Å^−3^

### 

Data collection: *APEX2* (Bruker, 2010 ▶); cell refinement: *SAINT* (Bruker, 2010 ▶); data reduction: *SAINT*; program(s) used to solve structure: *SHELXTL* (Sheldrick, 2008 ▶); program(s) used to refine structure: *SHELXTL*; molecular graphics: *SHELXTL*; software used to prepare material for publication: *SHELXTL*.

## Supplementary Material

Crystal structure: contains datablock(s) global, I. DOI: 10.1107/S1600536812026475/fy2058sup1.cif

Structure factors: contains datablock(s) I. DOI: 10.1107/S1600536812026475/fy2058Isup2.hkl

Supplementary material file. DOI: 10.1107/S1600536812026475/fy2058Isup3.cml

Additional supplementary materials:  crystallographic information; 3D view; checkCIF report

## Figures and Tables

**Table 1:** Hydrogen-bond geometry (Å, °)

*D*—H⋯*A*	*D*—H	H⋯*A*	*D*⋯*A*	*D*—H⋯*A*
O5—H5⋯O6^i^	0.86 (1)	1.81 (1)	2.6660 (16)	176 (2)

Symmetry code: (i) 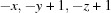.

**Fig. 1.**
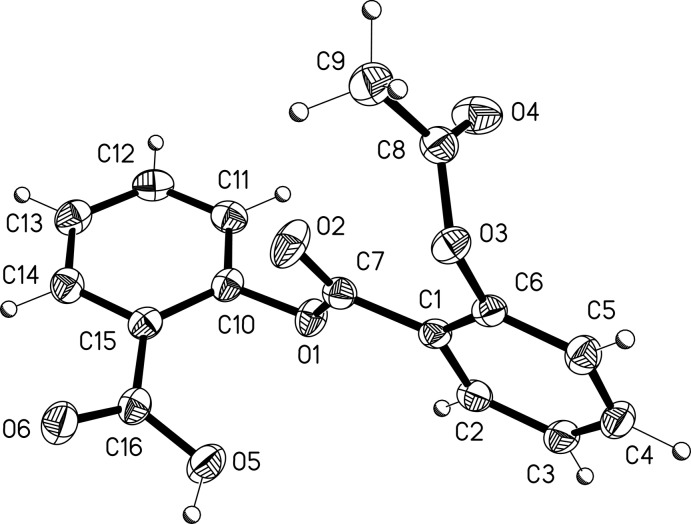
Molecular structure showing displacement ellipsoids at the 50% probability level for non-H atoms.

## supplementary crystallographic information

### Comment

Acetylsalicylsalicylic acid is a condensation (dehydration) product of acetylsalicylic acid (aspirin) and salicylic acid, and is a common impurity in commerical aspirin samples. Its pharmacological effects have been examined by Bundgaard (1974), and it has been suggested that the compound is a potentially immunogenic substance involved in the development of allergic reactions to aspirin.

### Experimental

The compound was prepared by acetylation of salicylsalicylic acid (purchased from Alfa Aesar) using acetic anhydride. 0.02 mol of salicylsalicylic acid was mixed with 0.01 mol of acetic anhydride with addition of 10% NaOH (5 ml) and *ca* 50 ml ice. The reactants were stirred for *ca* 2 h and the reaction was monitored by thin-layer chromotography. When the reaction was complete, the white solid was filtered and recrystallized from ethanol (yield 90%).

### Refinement

H atoms bound to C atoms were placed geometrically and allowed to ride during refinement with C—H = 0.95 (aromatic) or 0.98 Å (methyl) and with *U*_iso_(H) = 1.2 (aromatic) or 1.5*U*_eq_(C) (methyl). The H atom bound to O5 was located in a difference Fourier map and refined with an isotropic displacement parameter, with the O—H distance restrained to 0.85 (1) Å.

### Crystal data

**Table:**

C_16_H_12_O_6_	*F*(000) = 624
*M**_r_* = 300.26	*D*_x_ = 1.484 Mg m^−^^3^
Monoclinic, *P*2_1_/*c*	Mo *K*α radiation, λ = 0.71073 Å
Hall symbol: -P 2ybc	Cell parameters from 5582 reflections
*a* = 9.6314 (5) Å	θ = 2.9–25.0°
*b* = 7.7548 (3) Å	µ = 0.12 mm^−^^1^
*c* = 18.0763 (8) Å	*T* = 150 K
β = 95.572 (2)°	Lath, colourless
*V* = 1343.73 (11) Å^3^	0.40 × 0.20 × 0.02 mm
*Z* = 4	

### Data collection

**Table:**

Bruker Nonius X8 APEXII CCD diffractometer	2367 independent reflections
Radiation source: fine-focus sealed tube	1868 reflections with *I* > 2σ(*I*)
Graphite monochromator	*R*_int_ = 0.031
ω and φ scans	θ_max_ = 25.1°, θ_min_ = 3.6°
Absorption correction: multi-scan (*SADABS*; Sheldrick, 2003)	*h* = −11→11
*T*_min_ = 0.887, *T*_max_ = 0.998	*k* = −9→9
15807 measured reflections	*l* = −21→20

### Refinement

**Table:**

Refinement on *F*^2^	Primary atom site location: structure-invariant direct methods
Least-squares matrix: full	Secondary atom site location: difference Fourier map
*R*[*F*^2^ > 2σ(*F*^2^)] = 0.033	Hydrogen site location: inferred from neighbouring sites
*wR*(*F*^2^) = 0.080	H atoms treated by a mixture of independent and constrained refinement
*S* = 1.04	*w* = 1/[σ^2^(*F*_o_^2^) + (0.0341*P*)^2^ + 0.473*P*] where *P* = (*F*_o_^2^ + 2*F*_c_^2^)/3
2367 reflections	(Δ/σ)_max_ < 0.001
204 parameters	Δρ_max_ = 0.18 e Å^−^^3^
1 restraint	Δρ_min_ = −0.17 e Å^−^^3^

### Special details

**Table:**

Geometry. All e.s.d.'s (except the e.s.d. in the dihedral angle between two l.s. planes) are estimated using the full covariance matrix. The cell e.s.d.'s are taken into account individually in the estimation of e.s.d.'s in distances, angles and torsion angles; correlations between e.s.d.'s in cell parameters are only used when they are defined by crystal symmetry. An approximate (isotropic) treatment of cell e.s.d.'s is used for estimating e.s.d.'s involving l.s. planes.
Refinement. Refinement of *F*^2^ against ALL reflections. The weighted *R*-factor *wR* and goodness of fit *S* are based on *F*^2^, conventional *R*-factors *R* are based on *F*, with *F* set to zero for negative *F*^2^. The threshold expression of *F*^2^ > σ(*F*^2^) is used only for calculating *R*-factors(gt) *etc*. and is not relevant to the choice of reflections for refinement. *R*-factors based on *F*^2^ are statistically about twice as large as those based on *F*, and *R*- factors based on ALL data will be even larger.

### Fractional atomic coordinates and isotropic or equivalent isotropic displacement parameters (Å^2^)

**Table:**

	*x*	*y*	*z*	*U*_iso_*/*U*_eq_	
O1	0.25601 (10)	0.66416 (13)	0.32136 (5)	0.0262 (3)	
O2	0.38752 (12)	0.75630 (15)	0.42277 (7)	0.0369 (3)	
O3	0.62427 (10)	0.57401 (13)	0.46447 (5)	0.0245 (3)	
O4	0.69302 (12)	0.76366 (15)	0.38150 (6)	0.0367 (3)	
O5	0.07707 (13)	0.52229 (15)	0.41146 (6)	0.0373 (3)	
H5	0.052 (3)	0.451 (3)	0.4443 (11)	0.082 (8)*	
O6	0.00116 (13)	0.70903 (14)	0.49167 (6)	0.0351 (3)	
C1	0.45005 (15)	0.49405 (19)	0.36384 (8)	0.0212 (3)	
C2	0.40413 (16)	0.3714 (2)	0.31037 (8)	0.0249 (4)	
H2A	0.3206	0.3914	0.2791	0.030*	
C3	0.47842 (16)	0.2217 (2)	0.30243 (9)	0.0281 (4)	
H3A	0.4461	0.1398	0.2656	0.034*	
C4	0.59942 (17)	0.1906 (2)	0.34767 (9)	0.0296 (4)	
H4A	0.6494	0.0863	0.3426	0.036*	
C5	0.64823 (16)	0.3111 (2)	0.40048 (8)	0.0263 (4)	
H5A	0.7320	0.2903	0.4314	0.032*	
C6	0.57436 (15)	0.46175 (19)	0.40792 (8)	0.0213 (3)	
C7	0.36674 (15)	0.6510 (2)	0.37434 (8)	0.0232 (4)	
C8	0.67024 (15)	0.7322 (2)	0.44426 (9)	0.0266 (4)	
C9	0.68674 (17)	0.8504 (2)	0.50933 (9)	0.0331 (4)	
H9A	0.7316	0.9574	0.4953	0.050*	
H9B	0.7447	0.7949	0.5501	0.050*	
H9C	0.5948	0.8768	0.5254	0.050*	
C10	0.17065 (15)	0.8088 (2)	0.32687 (8)	0.0236 (4)	
C11	0.18395 (16)	0.9407 (2)	0.27723 (9)	0.0283 (4)	
H11A	0.2497	0.9319	0.2415	0.034*	
C12	0.10136 (16)	1.0862 (2)	0.27940 (9)	0.0305 (4)	
H12A	0.1091	1.1765	0.2445	0.037*	
C13	0.00762 (16)	1.1002 (2)	0.33224 (9)	0.0303 (4)	
H13A	−0.0480	1.2009	0.3343	0.036*	
C14	−0.00502 (16)	0.9675 (2)	0.38195 (9)	0.0273 (4)	
H14A	−0.0691	0.9784	0.4185	0.033*	
C15	0.07431 (15)	0.81807 (19)	0.37966 (8)	0.0234 (3)	
C16	0.04899 (16)	0.6788 (2)	0.43270 (9)	0.0258 (4)	

### Atomic displacement parameters (Å^2^)

**Table:**

	*U*^11^	*U*^22^	*U*^33^	*U*^12^	*U*^13^	*U*^23^
O1	0.0273 (6)	0.0265 (6)	0.0240 (6)	0.0042 (5)	−0.0015 (5)	−0.0055 (5)
O2	0.0365 (7)	0.0319 (7)	0.0394 (7)	0.0092 (5)	−0.0106 (5)	−0.0158 (6)
O3	0.0298 (6)	0.0216 (6)	0.0218 (5)	−0.0006 (5)	0.0007 (4)	−0.0024 (5)
O4	0.0456 (7)	0.0353 (7)	0.0296 (7)	−0.0121 (6)	0.0064 (5)	0.0006 (5)
O5	0.0572 (8)	0.0227 (7)	0.0343 (7)	0.0042 (6)	0.0154 (6)	0.0015 (5)
O6	0.0490 (7)	0.0281 (7)	0.0299 (6)	0.0074 (5)	0.0123 (5)	0.0022 (5)
C1	0.0256 (8)	0.0192 (8)	0.0196 (8)	−0.0030 (6)	0.0066 (6)	0.0000 (6)
C2	0.0259 (8)	0.0258 (9)	0.0235 (8)	−0.0048 (7)	0.0047 (6)	−0.0016 (7)
C3	0.0339 (9)	0.0233 (9)	0.0284 (9)	−0.0048 (7)	0.0097 (7)	−0.0078 (7)
C4	0.0372 (9)	0.0212 (9)	0.0322 (9)	0.0034 (7)	0.0125 (8)	−0.0008 (7)
C5	0.0297 (8)	0.0254 (9)	0.0246 (8)	0.0025 (7)	0.0056 (7)	0.0011 (7)
C6	0.0269 (8)	0.0203 (8)	0.0175 (8)	−0.0032 (6)	0.0062 (6)	−0.0006 (6)
C7	0.0230 (8)	0.0235 (8)	0.0233 (8)	−0.0025 (6)	0.0025 (7)	−0.0007 (7)
C8	0.0227 (8)	0.0262 (9)	0.0303 (9)	−0.0005 (7)	0.0004 (7)	−0.0002 (7)
C9	0.0333 (9)	0.0316 (10)	0.0341 (9)	−0.0021 (7)	0.0019 (7)	−0.0074 (8)
C10	0.0221 (7)	0.0239 (9)	0.0235 (8)	0.0008 (6)	−0.0042 (6)	−0.0055 (7)
C11	0.0274 (8)	0.0316 (10)	0.0253 (8)	−0.0048 (7)	−0.0001 (7)	−0.0007 (7)
C12	0.0326 (9)	0.0275 (9)	0.0299 (9)	−0.0044 (7)	−0.0052 (7)	0.0059 (7)
C13	0.0288 (9)	0.0249 (9)	0.0356 (10)	0.0045 (7)	−0.0044 (7)	0.0019 (8)
C14	0.0232 (8)	0.0287 (9)	0.0293 (8)	0.0022 (7)	−0.0003 (7)	−0.0015 (7)
C15	0.0231 (8)	0.0234 (8)	0.0228 (8)	−0.0009 (6)	−0.0032 (6)	−0.0024 (7)
C16	0.0250 (8)	0.0248 (9)	0.0271 (9)	0.0035 (7)	−0.0002 (7)	−0.0026 (7)

### Geometric parameters (Å, º)

**Table:**

O1—C7	1.3662 (18)	C5—C6	1.381 (2)
O1—C10	1.3998 (18)	C5—H5A	0.950
O2—C7	1.1995 (18)	C8—C9	1.488 (2)
O3—C8	1.3659 (19)	C9—H9A	0.980
O3—C6	1.3923 (18)	C9—H9B	0.980
O4—C8	1.2013 (18)	C9—H9C	0.980
O5—C16	1.3090 (19)	C10—C11	1.375 (2)
O5—H5	0.86 (1)	C10—C15	1.396 (2)
O6—C16	1.2242 (18)	C11—C12	1.383 (2)
C1—C6	1.395 (2)	C11—H11A	0.950
C1—C2	1.397 (2)	C12—C13	1.381 (2)
C1—C7	1.480 (2)	C12—H12A	0.950
C2—C3	1.378 (2)	C13—C14	1.379 (2)
C2—H2A	0.950	C13—H13A	0.950
C3—C4	1.378 (2)	C14—C15	1.391 (2)
C3—H3A	0.950	C14—H14A	0.950
C4—C5	1.385 (2)	C15—C16	1.480 (2)
C4—H4A	0.950		
			
C7—O1—C10	115.71 (11)	C8—C9—H9A	109.5
C8—O3—C6	117.58 (11)	C8—C9—H9B	109.5
C16—O5—H5	108.2 (16)	H9A—C9—H9B	109.5
C6—C1—C2	117.99 (14)	C8—C9—H9C	109.5
C6—C1—C7	121.35 (13)	H9A—C9—H9C	109.5
C2—C1—C7	120.63 (14)	H9B—C9—H9C	109.5
C3—C2—C1	120.84 (15)	C11—C10—C15	121.20 (14)
C3—C2—H2A	119.6	C11—C10—O1	117.20 (13)
C1—C2—H2A	119.6	C15—C10—O1	121.59 (14)
C2—C3—C4	120.23 (15)	C10—C11—C12	119.87 (14)
C2—C3—H3A	119.9	C10—C11—H11A	120.1
C4—C3—H3A	119.9	C12—C11—H11A	120.1
C3—C4—C5	120.08 (14)	C13—C12—C11	120.00 (15)
C3—C4—H4A	120.0	C13—C12—H12A	120.0
C5—C4—H4A	120.0	C11—C12—H12A	120.0
C6—C5—C4	119.63 (15)	C14—C13—C12	119.82 (15)
C6—C5—H5A	120.2	C14—C13—H13A	120.1
C4—C5—H5A	120.2	C12—C13—H13A	120.1
C5—C6—O3	117.13 (13)	C13—C14—C15	121.24 (14)
C5—C6—C1	121.20 (14)	C13—C14—H14A	119.4
O3—C6—C1	121.54 (13)	C15—C14—H14A	119.4
O2—C7—O1	121.63 (14)	C14—C15—C10	117.83 (14)
O2—C7—C1	126.80 (14)	C14—C15—C16	117.59 (13)
O1—C7—C1	111.55 (12)	C10—C15—C16	124.57 (14)
O4—C8—O3	121.91 (14)	O6—C16—O5	122.61 (14)
O4—C8—C9	127.32 (15)	O6—C16—C15	121.59 (14)
O3—C8—C9	110.77 (13)	O5—C16—C15	115.77 (13)
			
C6—C1—C2—C3	1.1 (2)	C6—O3—C8—O4	−14.2 (2)
C7—C1—C2—C3	−177.36 (13)	C6—O3—C8—C9	166.09 (12)
C1—C2—C3—C4	0.3 (2)	C7—O1—C10—C11	104.36 (15)
C2—C3—C4—C5	−1.2 (2)	C7—O1—C10—C15	−76.66 (17)
C3—C4—C5—C6	0.6 (2)	C15—C10—C11—C12	0.3 (2)
C4—C5—C6—O3	176.69 (13)	O1—C10—C11—C12	179.32 (13)
C4—C5—C6—C1	0.9 (2)	C10—C11—C12—C13	1.2 (2)
C8—O3—C6—C5	115.45 (14)	C11—C12—C13—C14	−1.1 (2)
C8—O3—C6—C1	−68.76 (17)	C12—C13—C14—C15	−0.6 (2)
C2—C1—C6—C5	−1.7 (2)	C13—C14—C15—C10	2.0 (2)
C7—C1—C6—C5	176.74 (13)	C13—C14—C15—C16	−177.04 (14)
C2—C1—C6—O3	−177.31 (12)	C11—C10—C15—C14	−1.9 (2)
C7—C1—C6—O3	1.1 (2)	O1—C10—C15—C14	179.14 (13)
C10—O1—C7—O2	0.9 (2)	C11—C10—C15—C16	177.09 (14)
C10—O1—C7—C1	179.84 (11)	O1—C10—C15—C16	−1.8 (2)
C6—C1—C7—O2	−6.4 (2)	C14—C15—C16—O6	−25.3 (2)
C2—C1—C7—O2	172.01 (15)	C10—C15—C16—O6	155.67 (15)
C6—C1—C7—O1	174.74 (12)	C14—C15—C16—O5	152.75 (14)
C2—C1—C7—O1	−6.87 (18)	C10—C15—C16—O5	−26.3 (2)

### Hydrogen-bond geometry (Å, º)

**Table:**

*D*—H···*A*	*D*—H	H···*A*	*D*···*A*	*D*—H···*A*
O5—H5···O6^i^	0.86 (1)	1.81 (1)	2.6660 (16)	176 (2)

Symmetry code: (i) −*x*, −*y*+1, −*z*+1.
